# The genome sequence of *Berberis vulgaris* L.

**DOI:** 10.12688/wellcomeopenres.23427.1

**Published:** 2024-12-03

**Authors:** Markus Ruhsam, Royal Botanic Garden Edinburgh Genome Acquisition Lab, Darwin Tree of Life Barcoding collective, Plant Genome Sizing collective, Wellcome Sanger Institute Tree of Life Management, Samples and Laboratory team, Wellcome Sanger Institute Scientific Operations: Sequencing Operations, Wellcome Sanger Institute Tree of Life Core Informatics team, Tree of Life Core Informatics collective, Darwin Tree of Life Consortium

**Affiliations:** 1 Royal Botanic Garden Edinburgh, Edinburgh, Scotland, UK

**Keywords:** Berberis vulgaris, common barberry, genome sequence, chromosomal, Ranunculales

## Abstract

We present a genome assembly from an individual *Berberis vulgaris* (Streptophyta; Magnoliopsida; Ranunculales; Berberidaceae). The genome sequence has a total length of 1,297.50 megabases. Most of the assembly is scaffolded into 14 chromosomal pseudomolecules. The mitochondrial and plastid genome assemblies have lengths of 786.62 kilobases and 166.26 kilobases, respectively.

## Species taxonomy

Eukaryota; Viridiplantae; Streptophyta; Streptophytina; Embryophyta; Tracheophyta; Euphyllophyta; Spermatophyta; Magnoliopsida; Mesangiospermae; Ranunculales; Berberidaceae; Berberidoideae; Berberideae; *Berberis*; *Berberis vulgaris* L. (NCBI:txid258209).

## Background

*Berberis vulgaris* (common barberry) is a spiny deciduous shrub up to 4 m tall which is native to most of Europe, parts of northwestern Africa and western Asia and has become naturalised in northern Europe and North America.

It functions as the alternate host for some rust fungus species in the genus *Puccinia* such as the dreaded wheat stem rust fungus ( *P. graminis*), a serious fungal disease of wheat ( Jin, 2011) and *P. arrhenatheri*, which causes the formation of witches' brooms on common barberry ( Naef *et al.*, 2002). Infected plants produce yellow discoloured leaves which emit a strong, flowery scent and sugary nectar, attracting insects by floral mimicry as sexual reproduction of the pathogen requires outcrossing by insects ( Naef *et al.*, 2002).

Since Linnaean times, *B. vulgaris* is known for its unusual rapid forward-snapping movement of the stamens once the filament basis of an individual stamen is touched by an insect ( Lechowski & Białczyk, 1992). This is likely to improve male reproductive success by accurately placing pollen on pollinators and decreasing foraging times of hit insects in flowers, thereby reducing nectar consumption and increasing pollen dispersal distances ( Li *et al.*, 2022).

*Berberis vulgaris* has also a long tradition as a medicinal plant for a wide range of diseases in Asia and Europe (reviewed in Imenshahidi & Hosseinzadeh, 2016). The main active ingredient is the alkaloid berberine which is supported for its antidiabetic, antiobesity, hypotensive and hypolipidemic properties by numerous studies ( Tabeshpour *et al.*, 2017).

*Berberis vulgaris* is a diploid species (2 *n* = 28) based on Czech ( Mesícek & Javurková-Jarolímová, 1992), Polish ( Pogan *et al.*, 1980) and Indian material ( Jeelani *et al.*, 2014). The first chromosome level genome of *B. vulgaris* may prove useful in investigating the responses to fungal infections as well as the medicinal properties of this species.

The genome of *Berberis vulgaris* was sequenced as part of the Darwin Tree of Life Project, a collaborative effort to sequence all named eukaryotic species in the Atlantic Archipelago of Britain and Ireland. Here we present a chromosomally complete genome sequence for *Berberis vulgaris*, based on a specimen from East of Drem, Scotland, United Kingdom.

## Genome sequence report

**Figure 1.**
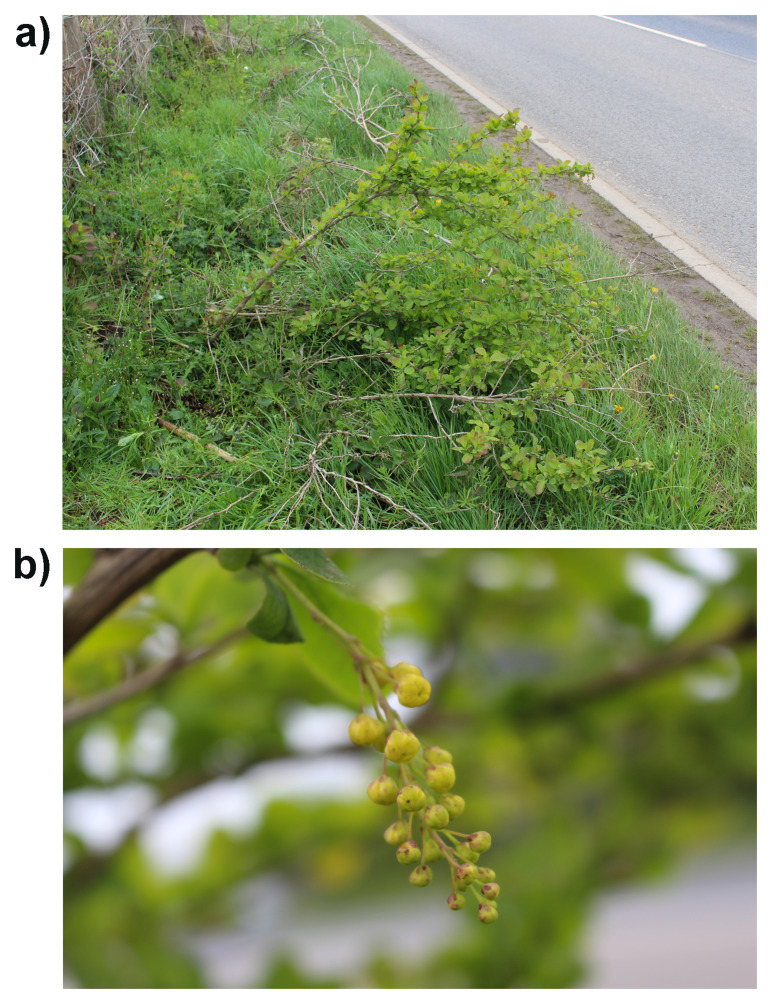
Photographs of the *Berberis vulgaris* (dmBerVulg1) specimen used for genome sequencing.

The genome of a specimen of *Berberis vulgaris* ( Figure 1) was sequenced using Pacific Biosciences single-molecule HiFi long reads, generating a total of 27.63 Gb (gigabases) from 1.86 million reads, providing approximately 28-fold coverage. Using flow cytometry, the genome size (1C-value) was estimated to be 1.64 pg, equivalent to 1,600 Mb. Primary assembly contigs were scaffolded with chromosome conformation Hi-C data, which produced 251.79 Gb from 1,667.46 million reads, yielding an approximate coverage of 194-fold. Specimen and sequencing information is summarised in Table 1.

**Table 1.** Specimen and sequencing data for *Berberis vulgaris*.

Project information			
**Study title**	Berberis vulgaris		
**Umbrella BioProject**	PRJEB60726		
**Species**	*Berberis vulgaris*		
**BioSample**	SAMEA10241407		
**NCBI taxonomy ID**	258209		
Specimen information			
**Technology**	**ToLID**	**BioSample accession**	**Organism part**
**PacBio long read sequencing**	dmBerVulg1	SAMEA10241495	leaf
**Hi-C sequencing**	dmBerVulg1	SAMEA10241495	leaf
Sequencing information			
**Platform**	**Run accession**	**Read count**	**Base count (Gb)**
**Hi-C Illumina NovaSeq 6000**	ERR11040197	1.54e+09	232.1
**Hi-C Illumina NovaSeq 6000**	ERR11040196	1.67e+09	251.79
**PacBio Sequel IIe**	ERR11029707	8.78e+05	13.29
**PacBio Sequel IIe**	ERR11029708	1.86e+06	27.63

**Figure 2.**
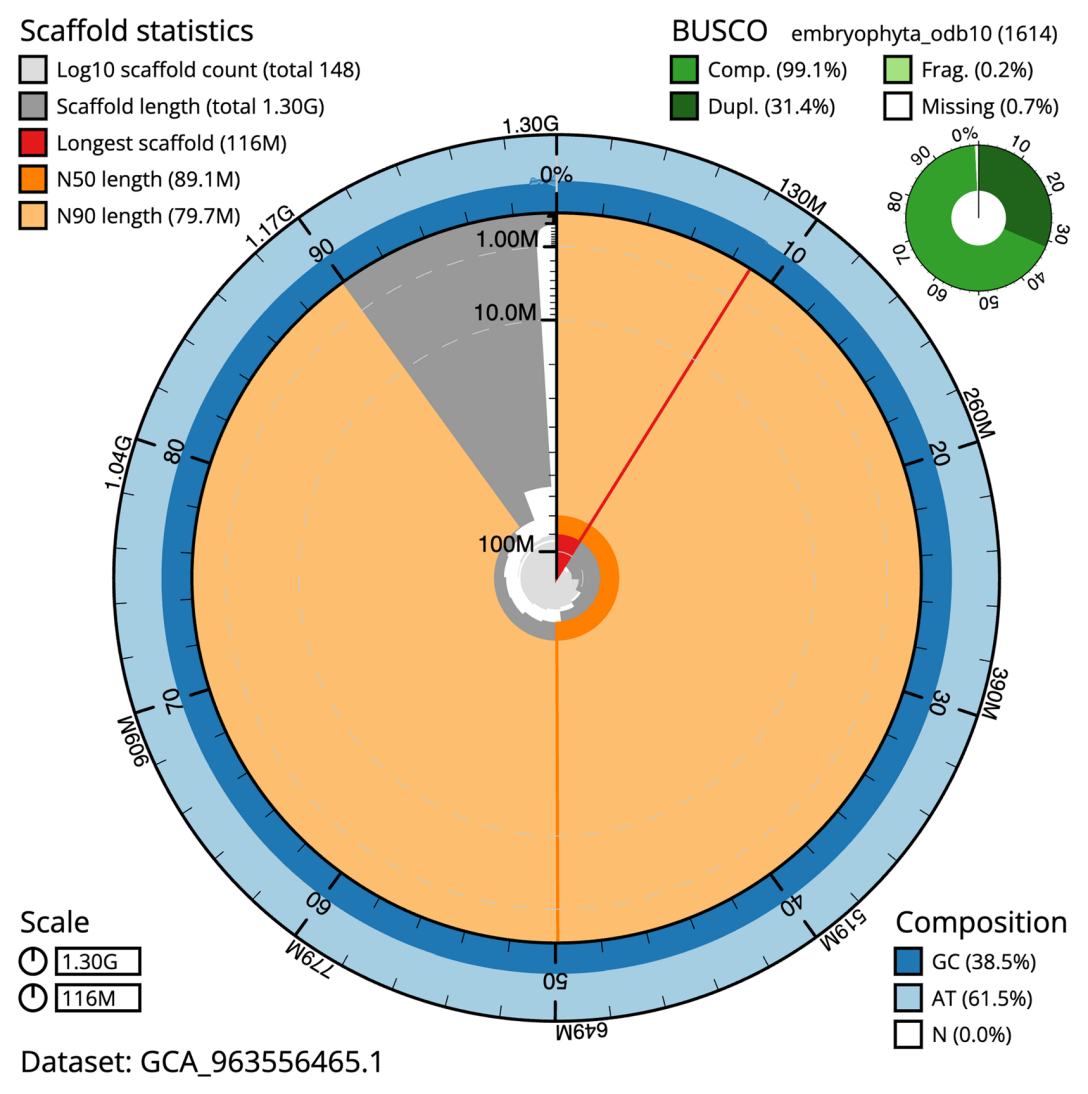
Snail plot summary of assembly statistics for assembly dmBerVulg1.1: The BlobToolKit snail plot shows N50 metrics and BUSCO gene completeness. The main plot is divided into 1,000 size-ordered bins around the circumference with each bin representing 0.1% of the 1,298,478,752 bp assembly. The distribution of scaffold lengths is shown in dark grey with the plot radius scaled to the longest scaffold present in the assembly (116,141,747 bp, shown in red). Orange and pale-orange arcs show the N50 and N90 scaffold lengths (89,131,704 and 79,668,399 bp), respectively. The pale grey spiral shows the cumulative scaffold count on a log scale with white scale lines showing successive orders of magnitude. The blue and pale-blue area around the outside of the plot shows the distribution of GC, AT and N percentages in the same bins as the inner plot. A summary of complete, fragmented, duplicated and missing BUSCO genes in the embryophyta_odb10 set is shown in the top right. An interactive version of this figure is available at https://blobtoolkit.genomehubs.org/view/GCA_963556465.1/dataset/GCA_963556465.1/snail.

**Figure 3.**
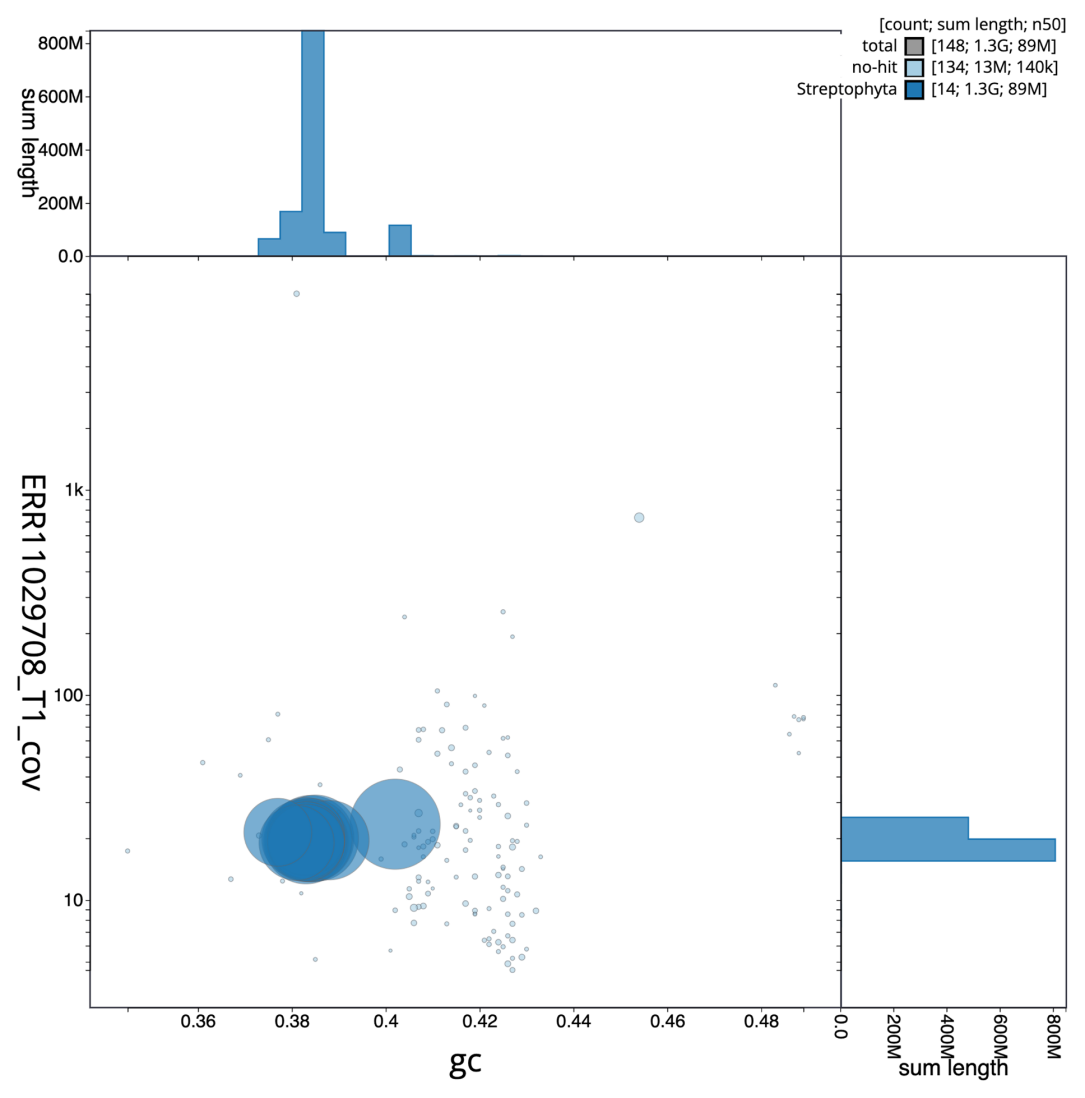
Blob plot of base coverage against GC proportion for sequences in the assembly dmBerVulg1.1: Scaffolds are coloured by phylum. Circles are sized in proportion to scaffold length. Histograms show the distribution of scaffold length sum along each axis. An interactive version of this figure is available at https://blobtoolkit.genomehubs.org/view/GCA_963556465.1/dataset/GCA_963556465.1/blob.

**Figure 4.**
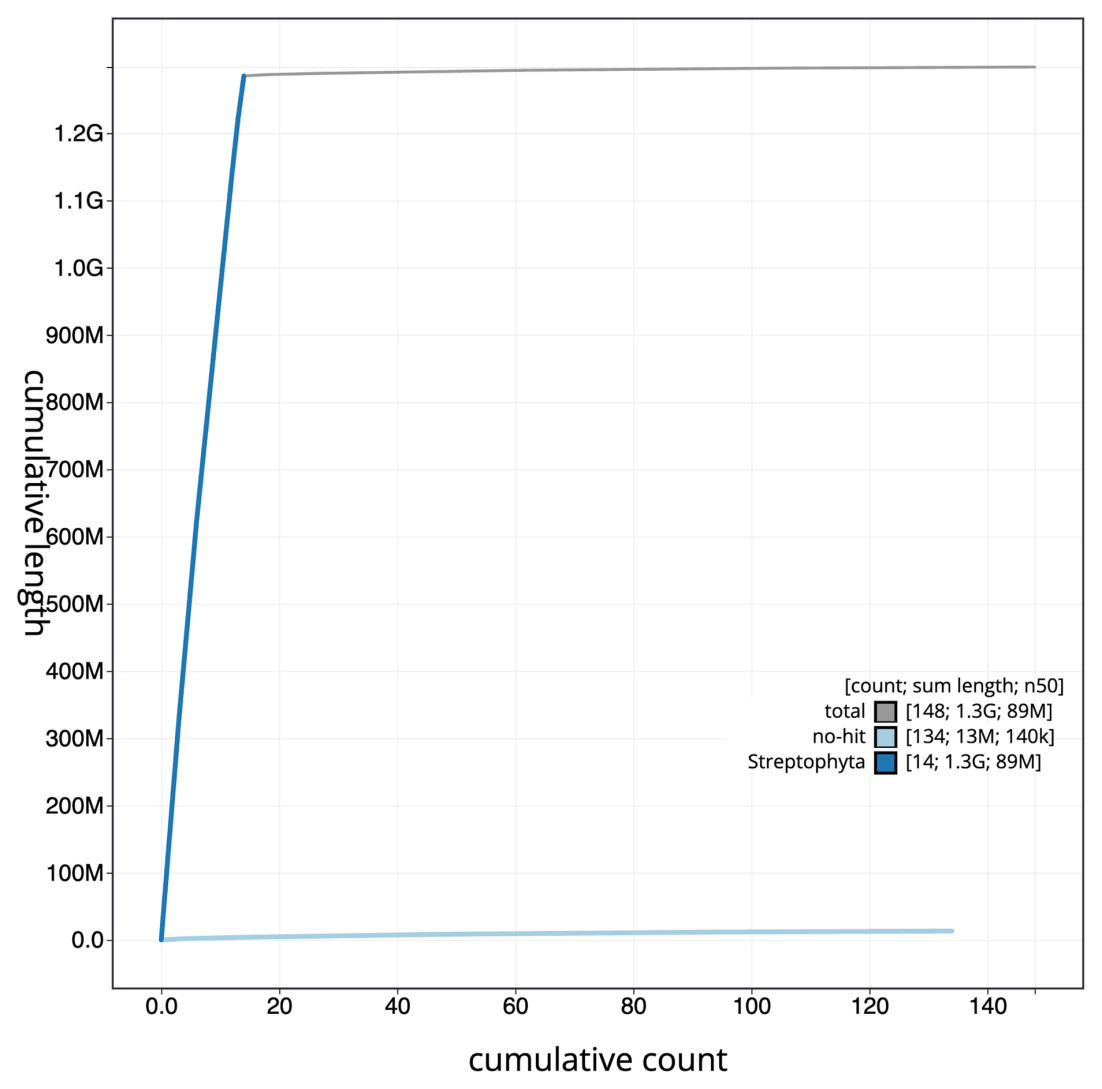
BlobToolKit cumulative sequence plot. The grey line shows cumulative length for all scaffolds. Coloured lines show cumulative lengths of scaffolds assigned to each phylum using the buscogenes taxrule. An interactive version of this figure is available at https://blobtoolkit.genomehubs.org/view/GCA_963556465.1/dataset/GCA_963556465.1/cumulative.

**Figure 5.**
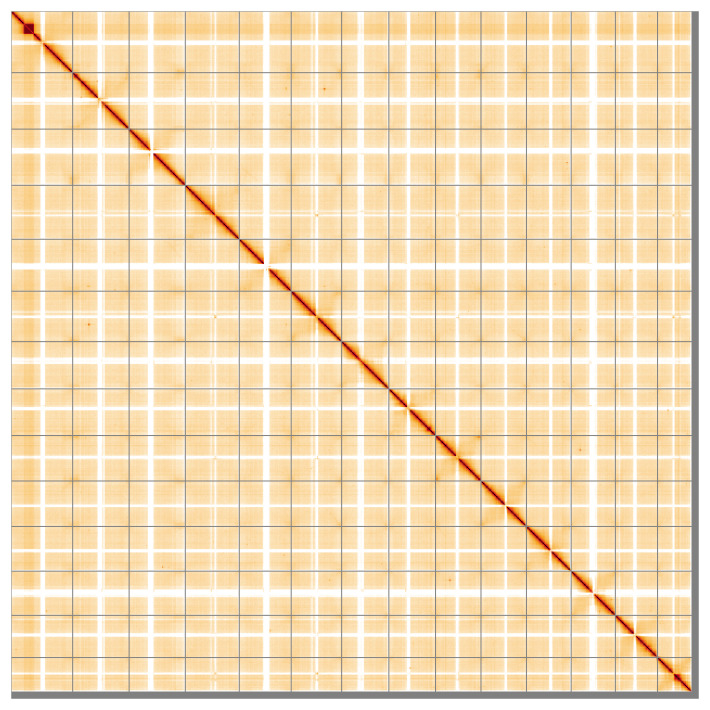
Genome assembly of *Berberis vulgaris*, dmBerVulg1.1: Hi-C contact map of the dmBerVulg1.1 assembly, visualised using HiGlass. Chromosomes are shown in order of size from left to right and top to bottom. An interactive version of this figure may be viewed at https://genome-note-higlass.tol.sanger.ac.uk/l/?d=PgFwmOONQjiHtQ4woAAIcg.

Manual assembly curation corrected 169 missing joins or mis-joins and 6 haplotypic duplications, reducing the scaffold number by 47.7%, and increasing the scaffold N50 by 9.78%. The final assembly has a total length of 1,297.50 Mb in 146 sequence scaffolds, with 721 gaps. The scaffold N50 is 89.1 Mb ( Table 2) The snail plot in Figure 2 provides a summary of the assembly statistics, while the distribution of assembly scaffolds on GC proportion and coverage is shown in Figure 3. The cumulative assembly plot in Figure 4 shows curves for subsets of scaffolds assigned to different phyla. Most (98.98%) of the assembly sequence was assigned to 14 chromosomal-level scaffolds. Chromosome-scale scaffolds confirmed by the Hi-C data are named in order of size ( Figure 5; Table 3). The exact order and orientation of the centromeric repeat regions is unknown. While not fully phased, the assembly deposited is of one haplotype. Contigs corresponding to the second haplotype have also been deposited. The mitochondrial and plastid genomes were also assembled and can be found as contigs within the multifasta file of the genome submission.

**Table 2.** Genome assembly data for *Berberis vulgaris*, dmBerVulg1.1.

Genome assembly		
Assembly name	dmBerVulg1.1	
Assembly accession	GCA_963556465.1	
*Accession of alternate haplotype*	*GCA_963556485.1*	
Span (Mb)	1,297.50	
Number of contigs	869	
Number of scaffolds	146	
Longest scaffold (Mb)	116.14	
Assembly metrics *		*Benchmark*
Contig N50 length (Mb)	2.9	*≥ 1 Mb*
Scaffold N50 length (Mb)	89.1	*= chromosome N50*
Consensus quality (QV)	62.9	*≥ 40*
*k*-mer completeness	100.0%	*≥ 95%*
BUSCO **	C:98.9%[S:67.3%,D:31.6%], F:0.4%,M:0.7%,n:1,614	*S > 90%, D < 5%*
Percentage of assembly mapped to chromosomes	98.98%	*≥ 90%*
Organelles	Mitochondrial genome: 786.62 kb Plastid genome: 166.26 kb	*complete single alleles*

* Assembly metric benchmarks are adapted from column VGP-2020 of “Table 1: Proposed standards and metrics for defining genome assembly quality” from Rhie *et al.* (2021). ** BUSCO scores based on the embryophyta_odb10 BUSCO set using version 5.4.3. C = complete [S = single copy, D = duplicated], F = fragmented, M = missing, n = number of orthologues in comparison. A full set of BUSCO scores is available at https://blobtoolkit.genomehubs.org/view/Berberis_vulgaris/dataset/GCA_963556465.1/busco.

**Table 3.** Chromosomal pseudomolecules in the genome assembly of *Berberis vulgaris*, dmBerVulg1.

INSDC accession	Name	Length (Mb)	GC%
OY747124.1	1	116.14	40.0
OY747125.1	2	106.47	38.5
OY747126.1	3	106.09	38.5
OY747127.1	4	101.74	38.5
OY747128.1	5	98.16	38.5
OY747129.1	6	95.13	38.5
OY747130.1	7	89.13	39.0
OY747131.1	8	88.01	38.0
OY747132.1	9	86.17	38.5
OY747133.1	10	85.62	38.5
OY747134.1	11	84.46	38.5
OY747135.1	12	83.66	38.5
OY747136.1	13	79.67	38.0
OY747137.1	14	64.78	37.5
OY747138.1	MT	0.79	45.5
OY747139.1	Pltd	0.17	38.0

The estimated Quality Value (QV) of the final assembly is 62.9 with *k*-mer completeness of 100.0%, and the assembly has a BUSCO v5.4.3 completeness of 98.9% (single = 67.3%, duplicated = 31.6%), using the embryophyta_odb10 reference set ( *n* = 1,614).

Metadata for specimens, BOLD barcode results, spectra estimates, sequencing runs, contaminants and pre-curation assembly statistics are given at https://links.tol.sanger.ac.uk/species/258209.

## Methods

### Sample acquisition, DNA barcoding and genome size estimation

Leaf material of *Berberis vulgaris* (specimen ID EDTOL01214, ToLID dmBerVulg1) was collected from East of Drem, Scotland, United Kingdom (latitude 56.01, longitude –2.74) on 2021-05-13. The specimen was collected and identified by Markus Ruhsam (Royal Botanic Garden Edinburgh) and preserved in liquid nitrogen. The herbarium voucher associated with the sequenced plant is https://data.rbge.org.uk/herb/E01152236 and is deposited in the herbarium of RBG Edinburgh (E).

The initial species identification was verified by an additional DNA barcoding process according to the framework developed by Twyford *et al.* (2024). Part of the plant specimen was preserved in silica gel desiccant. A DNA extraction from the dried plant was amplified by PCR for standard barcode markers, with the amplicons sequenced and compared to public sequence databases including GenBank and the Barcode of Life Database (BOLD). The barcode sequences for this specimen are openly available on BOLD ( Ratnasingham & Hebert, 2007). Following whole genome sequence generation, DNA barcodes were also used alongside the initial barcoding data for sample tracking through the genome production pipeline at the Wellcome Sanger Institute ( Twyford *et al.*, 2024). The standard operating procedures for the Darwin Tree of Life barcoding have been deposited on protocols.io ( Beasley *et al.*, 2023).

The genome size was estimated by flow cytometry using the fluorochrome propidium iodide and following the ‘one-step’ method as outlined in Pellicer *et al.* (2021). For this species, the General Purpose Buffer (GPB) supplemented with 3% PVP and 0.08% (v/v) beta-mercaptoethanol was used for isolation of nuclei ( Loureiro *et al.*, 2007), and the internal calibration standard was Pisum sativum ‘Ctirad’ with an assumed 1C-value of 4,445 Mb ( Doležel *et al.*, 1998).

### Nucleic acid extraction

The workflow for high molecular weight (HMW) DNA extraction at the WSI Tree of Life Core Laboratory includes a sequence of core procedures: sample preparation and homogenisation, DNA extraction, fragmentation and purification. Detailed protocols are available on protocols.io ( Denton *et al.*, 2023). In sample preparation, the dmBerVulg1 sample was weighed and dissected on dry ice ( Jay *et al.*, 2023). For sample homogenisation, leaf tissue was cryogenically disrupted using the Covaris cryoPREP ^®^ Automated Dry Pulverizer ( Narváez-Gómez *et al.*, 2023). HMW DNA was extracted using the Automated Plant MagAttract v2 protocol ( Todorovic *et al.*, 2023). HMW DNA was sheared into an average fragment size of 12–20 kb in a Megaruptor 3 system ( Bates *et al.*, 2023). Sheared DNA was purified by solid-phase reversible immobilisation, using AMPure PB beads to eliminate shorter fragments and concentrate the DNA ( Strickland *et al.*, 2023). The concentration of the sheared and purified DNA was assessed using a Nanodrop spectrophotometer and Qubit Fluorometer and Qubit dsDNA High Sensitivity Assay kit. Fragment size distribution was evaluated by running the sample on the FemtoPulse system.

### Library preparation and sequencing

Pacific Biosciences HiFi circular consensus DNA sequencing libraries were constructed according to the manufacturers’ instructions. DNA sequencing was performed by the Scientific Operations core at the WSI on a Pacific Biosciences Sequel IIe instrument.

Hi-C data were generated from the leaf tissue of dmBerVulg1 using the Arima-HiC v2 kit. Tissue was finely ground using cryoPrep and then subjected to nuclei isolation using the Qiagen QProteome Kit. After isolation, the nuclei were fixed, and the DNA crosslinked using pure formaldehyde. The crosslinked DNA was then digested using a restriction enzyme master mix. The 5’-overhangs were filled in and labelled with a biotinylated nucleotide, followed by proximity ligation. The biotinylated DNA constructs were fragmented to a size of 400 to 600 bp using a Covaris E220 sonicator. The DNA was then enriched, barcoded, and amplified using the NEBNext Ultra II DNA Library Prep Kit, following the manufacturer's instructions. Hi-C sequencing was performed using paired-end sequencing with a read length of 150 bp on an Illumina NovaSeq 6000 instrument.

### Genome assembly, curation and evaluation

***Assembly***

The HiFi reads were first assembled using Hifiasm ( Cheng *et al.*, 2021) with the --primary option. Haplotypic duplications were identified and removed using purge_dups ( Guan *et al.*, 2020). The Hi-C reads were mapped to the primary contigs using bwa-mem2 ( Vasimuddin *et al.*, 2019). The contigs were further scaffolded using the provided Hi-C data ( Rao *et al.*, 2014) in YaHS ( Zhou *et al.*, 2023) using the --break option. The scaffolded assemblies were evaluated using Gfastats ( Formenti *et al.*, 2022), BUSCO ( Manni *et al.*, 2021) and MERQURY.FK ( Rhie *et al.*, 2020). The organelle genomes were assembled using OATK ( Zhou, 2023).

***Curation***

The assembly was decontaminated using the Assembly Screen for Cobionts and Contaminants (ASCC) pipeline (article in preparation). Flat files and maps used in curation were generated in TreeVal ( Pointon *et al.*, 2023). Manual curation was primarily conducted using PretextView ( Harry, 2022), with additional insights provided by JBrowse2 ( Diesh *et al.*, 2023) and HiGlass ( Kerpedjiev *et al.*, 2018). Scaffolds were visually inspected and corrected as described by Howe *et al.* (2021). Any identified contamination, missed joins, and mis-joins were corrected, and duplicate sequences were tagged and removed. The process is documented at https://gitlab.com/wtsi-grit/rapid-curation (article in preparation).

***Evaluation of final assembly***

The final assembly was post-processed and evaluated using the three Nextflow ( Di Tommaso *et al.*, 2017) DSL2 pipelines: sanger-tol/readmapping ( Surana *et al.*, 2023a), sanger-tol/genomenote ( Surana *et al.*, 2023b), and sanger-tol/blobtoolkit ( Muffato *et al.*, 2024). The readmapping pipeline aligns the Hi-C reads using bwa-mem2 ( Vasimuddin *et al.*, 2019) and combines the alignment files with SAMtools ( Danecek *et al.*, 2021). The genomenote pipeline converts the Hi-C alignments into a contact map using BEDTools ( Quinlan & Hall, 2010) and the Cooler tool suite ( Abdennur & Mirny, 2020). The contact map is visualised in HiGlass ( Kerpedjiev *et al.*, 2018). This pipeline also computes *k*-mer completeness and QV consensus quality values with FastK and MERQURY.FK, and runs BUSCO ( Manni *et al.*, 2021) to assess completeness.

The blobtoolkit pipeline is a Nextflow port of the previous Snakemake Blobtoolkit pipeline ( Challis *et al.*, 2020). It aligns the PacBio reads in SAMtools and minimap2 ( Li, 2018) and generates coverage tracks for regions of fixed size. In parallel, it queries the GoaT database ( Challis *et al.*, 2023) to identify all matching BUSCO lineages to run BUSCO ( Manni *et al.*, 2021). For the three domain-level BUSCO lineages, the pipeline aligns the BUSCO genes to the UniProt Reference Proteomes database ( Bateman *et al.*, 2023) with DIAMOND ( Buchfink *et al.*, 2021) blastp. The genome is also split into chunks according to the density of the BUSCO genes from the closest taxonomic lineage, and each chunk is aligned to the UniProt Reference Proteomes database with DIAMOND blastx. Genome sequences without a hit are chunked with seqtk and aligned to the NT database with blastn ( Altschul *et al.*, 1990). The blobtools suite combines all these outputs into a blobdir for visualisation.

The genome evaluation pipelines were developed using nf-core tooling ( Ewels *et al.*, 2020) and MultiQC ( Ewels *et al.*, 2016), relying on the Conda package manager, the Bioconda initiative ( Grüning *et al.*, 2018), the Biocontainers infrastructure ( da Veiga Leprevost *et al.*, 2017), as well as the Docker ( Merkel, 2014) and Singularity ( Kurtzer *et al.*, 2017) containerisation solutions.

Table 4 contains a list of relevant software tool versions and sources.

**Table 4.** Software tools: versions and sources.

Software tool	Version	Source
BEDTools	2.30.0	https://github.com/arq5x/bedtools2
BLAST	2.14.0	ftp://ftp.ncbi.nlm.nih.gov/blast/executables/blast+/
BlobToolKit	4.3.7	https://github.com/blobtoolkit/blobtoolkit
BUSCO	5.4.3 and 5.5.0	https://gitlab.com/ezlab/busco
bwa-mem2	2.2.1	https://github.com/bwa-mem2/bwa-mem2
Cooler	0.8.11	https://github.com/open2c/cooler
DIAMOND	2.1.8	https://github.com/bbuchfink/diamond
fasta_windows	0.2.4	https://github.com/tolkit/fasta_windows
FastK	427104ea91c78c3b8b8b49f1a7d6bbeaa869ba1c	https://github.com/thegenemyers/FASTK
Gfastats	1.3.6	https://github.com/vgl-hub/gfastats
GoaT CLI	0.2.5	https://github.com/genomehubs/goat-cli
Hifiasm	0.16.1-r375	https://github.com/chhylp123/hifiasm
HiGlass	44086069ee7d4d3f6f3f0012569789ec138f42b84aa44357826c0b6753eb28de	https://github.com/higlass/higlass
Merqury.FK	d00d98157618f4e8d1a9190026b19b471055b22e	https://github.com/thegenemyers/MERQURY.FK
MitoHiFi	2	https://github.com/marcelauliano/MitoHiFi
MultiQC	1.14, 1.17, and 1.18	https://github.com/MultiQC/MultiQC
NCBI Datasets	15.12.0	https://github.com/ncbi/datasets
Nextflow	23.04.0-5857	https://github.com/nextflow-io/nextflow
PretextView	0.2	https://github.com/sanger-tol/PretextView
OATK	0.2	https://github.com/c-zhou/oatk
purge_dups	1.2.5	https://github.com/dfguan/purge_dups
samtools	1.16.1, 1.17, and 1.18	https://github.com/samtools/samtools
sanger-tol/genomenote	1.1.1	https://github.com/sanger-tol/genomenote
sanger-tol/readmapping	1.2.1	https://github.com/sanger-tol/readmapping
Seqtk	1.3	https://github.com/lh3/seqtk
Singularity	3.9.0	https://github.com/sylabs/singularity
TreeVal	1.0.0	https://github.com/sanger-tol/treeval
YaHS	1.2a.2	https://github.com/c-zhou/yahs

### Wellcome Sanger Institute – Legal and Governance

The materials that have contributed to this genome note have been supplied by a Darwin Tree of Life Partner. The submission of materials by a Darwin Tree of Life Partner is subject to the **‘Darwin Tree of Life Project Sampling Code of Practice’**, which can be found in full on the Darwin Tree of Life website here. By agreeing with and signing up to the Sampling Code of Practice, the Darwin Tree of Life Partner agrees they will meet the legal and ethical requirements and standards set out within this document in respect of all samples acquired for, and supplied to, the Darwin Tree of Life Project.

Further, the Wellcome Sanger Institute employs a process whereby due diligence is carried out proportionate to the nature of the materials themselves, and the circumstances under which they have been/are to be collected and provided for use. The purpose of this is to address and mitigate any potential legal and/or ethical implications of receipt and use of the materials as part of the research project, and to ensure that in doing so we align with best practice wherever possible. The overarching areas of consideration are:

•    Ethical review of provenance and sourcing of the material

•    Legality of collection, transfer and use (national and international)

Each transfer of samples is further undertaken according to a Research Collaboration Agreement or Material Transfer Agreement entered into by the Darwin Tree of Life Partner, Genome Research Limited (operating as the Wellcome Sanger Institute), and in some circumstances other Darwin Tree of Life collaborators.

## Data Availability

European Nucleotide Archive: Berberis vulgaris. Accession number PRJEB60726; https://identifiers.org/ena.embl/PRJEB60726. The genome sequence is released openly for reuse. The *Berberis vulgaris* genome sequencing initiative is part of the Darwin Tree of Life (DToL) project. All raw sequence data and the assembly have been deposited in INSDC databases. The genome will be annotated using available RNA-Seq data and presented through the Ensembl pipeline at the European Bioinformatics Institute. Raw data and assembly accession identifiers are reported in Table 1.

## Author information

Members of the Royal Botanic Garden Edinburgh Genome Acquisition Lab are listed here: https://doi.org/10.5281/zenodo.4786682.

Members of the Plant Genome Sizing collective are listed here: https://doi.org/10.5281/zenodo.7994306.

Members of the Darwin Tree of Life Barcoding collective are listed here: https://doi.org/10.5281/zenodo.12158331

Members of the Wellcome Sanger Institute Tree of Life Management, Samples and Laboratory team are listed here: https://doi.org/10.5281/zenodo.12162482.

Members of Wellcome Sanger Institute Scientific Operations: Sequencing Operations are listed here: https://doi.org/10.5281/zenodo.12165051.

Members of the Wellcome Sanger Institute Tree of Life Core Informatics team are listed here: https://doi.org/10.5281/zenodo.12160324.

Members of the Tree of Life Core Informatics collective are listed here: https://doi.org/10.5281/zenodo.12205391.

Members of the Darwin Tree of Life Consortium are listed here: https://doi.org/10.5281/zenodo.4783558.
