# Supplementary material for: The genome sequence of Berberis vulgaris L
Source: Wellcome Open Res. 2024 Dec 3;9:710. [Version 1] doi: 10.12688/wellcomeopenres.23427.1 (PMC11803380; doi:10.12688/wellcomeopenres.23427.1)
Supplement: Peer review history

# Reviewer report: Reviewer response for version 1

**DOI:** 10.21956/wellcomeopenres.25837.r115632

**Published:** 2025-01-02

**Authors:** Peng-Fei Ma

**Affiliations:** 1 Germplasm Bank of Wild Species & Yunnan Key Laboratory of Crop Wild Relatives Omics, Kunming Institute of Botany, Chinese Academy of Sciences, Kunming, China

The study has reported a high-quality reference genome assembly of common barberry.  This plant is well known for being as host of rust fungus, as well as a medicinal plant in Asia and Europe. The genomic data generated here will serve as genetic resource for studying this important plant in future.

Are sufficient details of methods and materials provided to allow replication by others?

Yes

Is the rationale for creating the dataset(s) clearly described?

Yes

Are the datasets clearly presented in a useable and accessible format?

Yes

Are the protocols appropriate and is the work technically sound?

Yes

Reviewer Expertise:

Plant genomics and evolution.

I confirm that I have read this submission and believe that I have an appropriate level of expertise to confirm that it is of an acceptable scientific standard.

# Reviewer report: Reviewer response for version 1

**DOI:** 10.21956/wellcomeopenres.25837.r115628

**Published:** 2024-12-31

**Authors:** Murukarthick Jayakodi

**Affiliations:** 1 Texas A&M University, College Station, Texas, USA

Ruhsam et al. report the genome sequence of common barberry ( *Berberis vulgaris*) which is a diploid (n=19) with a genome size of ~1.6 Gb.  They used long read technology of pacbio Hifi and Hi-C to build chromosome-level assembly. The assembly validation with known programs showed good assembly completeness and gene space. Overall, this reference genome could serve as a useful resource for gene mapping and comparative studies. Before indexing, the authors are asked to provide the heterozygosity information of common barberry.   And, future direction towards annotating repeats and genes.

Are sufficient details of methods and materials provided to allow replication by others?

Yes

Is the rationale for creating the dataset(s) clearly described?

Yes

Are the datasets clearly presented in a useable and accessible format?

Yes

Are the protocols appropriate and is the work technically sound?

Yes

Reviewer Expertise:

Plant Genomics

I confirm that I have read this submission and believe that I have an appropriate level of expertise to confirm that it is of an acceptable scientific standard.
